# Prevalence of right ventricular dysfunction and impact on all-cause death in hospitalized patients with COVID-19: a systematic review and meta-analysis

**DOI:** 10.1038/s41598-021-96955-8

**Published:** 2021-09-07

**Authors:** Bernadette Corica, Alberto Maria Marra, Stefania Basili, Roberto Cangemi, Antonio Cittadini, Marco Proietti, Giulio Francesco Romiti

**Affiliations:** 1 Department of Translational and Precision Medicine, Sapienza – University of Rome, Rome, Italy; 2 Department of Translational Medical Sciences, “Federico II” University of Naples, Naples, Italy; 3 Center for Pulmonary Hypertension, Thoraxklinik at Heidelberg University Hospital, Heidelberg, Germany; 4 Department of Clinical Sciences and Community Health, University of Milan, Milan, Italy; 5 Geriatric Unit, IRCCS Istituti Clinici Scientifici Maugeri, Via Camaldoli 64, 20138 Milan, Italy; 6 Liverpool Centre for Cardiovascular Science, University of Liverpool and Liverpool Heart & Chest Hospital, Liverpool, UK

**Keywords:** Cardiology, Cardiovascular diseases, Heart failure, Ultrasonography

## Abstract

The Coronavirus Disease (COVID-19) pandemic imposed a high burden of morbidity and mortality. In COVID-19, direct lung parenchymal involvement and pulmonary microcirculation dysfunction may entail pulmonary hypertension (PH). PH and direct cardiac injury beget right ventricular dysfunction (RVD) occurrence, which has been frequently reported in COVID-19 patients; however, the prevalence of RVD and its impact on outcomes during COVID-19 are still unclear. This study aims to evaluate the prevalence of RVD and associated outcomes in patients with COVID-19, through a Systematic Review and Meta-Analysis. MEDLINE and EMBASE were systematically searched from inception to 15th July 2021. All studies reporting either the prevalence of RVD in COVID-19 patients or all-cause death according to RVD status were included. The pooled prevalence of RVD and Odds Ratio (OR) for all-cause death according to RVD status were computed and reported. Subgroup analysis and meta-regression were also performed. Among 29 studies (3813 patients) included, pooled prevalence of RVD was 20.4% (95% CI 17.1–24.3%; 95% PI 7.8–43.9%), with a high grade of heterogeneity. No significant differences were found across geographical locations, or according to the risk of bias. Severity of COVID-19 was associated with increased prevalence of RVD at meta-regression. The presence of RVD was found associated with an increased likelihood of all-cause death (OR 3.32, 95% CI 1.94–5.70). RVD was found in 1 out of 5 COVID-19 patients, and was associated with all-cause mortality. RVD may represent one crucial marker for prognostic stratification in COVID-19; further prospective and larger are needed to investigate specific management and therapeutic approach for these patients.

## Introduction

Severe acute respiratory syndrome coronavirus 2 (SARS-CoV-2) and its associated disease (COVID-19), plagued the world during 2020, with the World Health Organization declaring a pandemic state earlier in the year^1^. By 13th July 2021, an estimated 4 million deaths were attributed to COVID-19 worldwide^2^, with an extremely high healthcare, social, and economic burden. Most of the disease’s mortality and severity have been attributed to respiratory complications of the disease; indeed, patients may develop severe pneumonia up to Acute Respiratory Distress Syndrome (ARDS). Beyond direct alveolar involvement, also lung microcirculation seems to be affected in these patients. Autopsies studies revealed a pattern of pulmonary endothelial dysfunction with increased inflammatory infiltrate and microvascular thrombosis^3^. All the conditions mentioned above may lead to the development of increased pulmonary pressures and right ventricle overload.

On the other hand, cardiovascular complications have been early addressed as one concern in these patients^4^. While several factors may influence the severity and clinical course of the disease^5^, cardiovascular disease, including arrhythmia, myocardial disfunction and myocardial injury, have been repeatedly identified as potential key detrimental factors in patients with COVID-19^6–8^. Lung parenchymal involvement, pulmonary microvascular pathologic changes, right ventricular pressure overload, and direct myocardial injury exert a synergic detrimental effect on the right ventricular function.

Indeed, right ventricular dysfunction (RVD)^9^ has been described as a potential predictor of poor outcomes in small preliminary studies, but its prevalence and associated outcomes in patients with COVID-19 are far from being elucidated. Clarification of the prevalence of RVD, and its associated outcomes in patients with COVID-19, may promote the implementation of tailored strategies for the screening, prevention, and treatment of right ventricular impairment.

Amid this pandemic, systematic review and meta-analysis have been depicted as essential tools to provide a timely and comprehensive synthesis of evidence during the COVID-19 pandemic^10^. Moving from this, this systematic review and meta-analysis aimed to estimate the prevalence of RVD among patients with COVID-19 and to explore its impact on all-cause mortality.

## Materials and methods

This systematic review has been performed according to the Preferred Reporting Items for Systematic Reviews and Meta-Analyses (PRISMA) guidelines and recommendations. The protocol was registered into the international register of systematic reviews PROSPERO, N. CRD42021227946.

### Search strategy

Systematic and comprehensive literature research was performed on MEDLINE and EMBASE databases, from inception to 15th July 2021. The search strategy included a combination of terms related to the research question, including ‘right ventricular dysfunction’, ‘COVID-19’, and ‘SARS-CoV-2’. The full search strategy is available in Supplementary Material, Table S1.

### Study selection

All records retrieved from the database search were systematically assessed by two independent authors (BC and GFR) according to titles and abstracts; articles included after this phase were subsequently screened for full-text eligibility. Study selection was performed with the use of a standardized web-based platform (Covidence). Any disagreement during each phase was discussed collegially. Inclusion criteria were: (i) any study reporting the prevalence of RVD; or (ii) any study reporting outcomes in patients with COVID-19 according to the RVD status (i.e., number of patients with and without RVD who died). Exclusion criteria were: (i) case reports, conference abstracts, editorial, comments, systematic reviews, meta-analyses and guidelines, (ii) studies that enrolled less than 30 COVID-19 patients, and (iii) articles in languages other than English, Italian or Spanish.

References of the included studies were additionally searched for other relevant articles that were not retrieved from the literature search. In the case of two or more studies based on the same cohort of subjects and exploring the same outcome(s), only the most recently published were selected and included.

### Outcomes definition

Primary outcomes were defined as (i) prevalence of RVD, as defined in the original studies, and (ii) all-cause death according to RVD status. When RVD was not clearly defined in the original studies or multiple definitions were reported, we considered RVD according to the study-defined TAPSE cut-off, if available, to increase the homogeneity of RVD definition among studies included. Among the “all-cause death” definition, we also included the in-hospital mortality and 30-day mortality, as defined in the original studies.

### Data extraction and quality assessment

Data from the studies included were independently extracted by two co-authors (BC and GFR) using a standardized electronic form. Data on sample size, number of individuals with and without RVD, mortality, and follow-up time were extracted. We collected data about study design and cohort baseline characteristics (i.e., age, sex, associated comorbidities including hypertension, diabetes mellitus, congestive heart failure (CHF)), and data on the severity of the disease or intensity of care received (i.e. intensive care unit (ICU), mechanically ventilated patients), when available. Proportion of patients with severe COVID-19 disease enrolled was also extracted; we defined severity according to the original studies definitions, when available, or according to a diagnosis of ARDS, or need for mechanical ventilation.

All studies included were independently evaluated by two co-authors (BC and GFR) to evaluate their quality and assess the risk of bias. We assessed the risk of bias separately for the two primary outcomes of the study. For studies reporting the prevalence of RVD, we used a customized version of the Newcastle–Ottawa Scale (NOS) for cross-sectional studies, composed of 5 items across 3 domains (selection, comparability, outcomes), with a maximum of 5 points. Each study with a score ≤ 3 was considered at high risk of bias. We used a customized version of the NOS for cohort studies for studies reporting outcomes, composed of 8 items across 3 domains (selection, comparability, outcomes), with a maximum of 9 points. Each study with a score ≤ 6 was considered at high risk of bias.

Publication bias was assessed for studies reporting outcomes according to RVD status. Funnel plots were visually inspected for asymmetricity; furthermore, Egger’s test was also performed and reported.

### Statistical analysis

Pooled prevalence of RVD, 95% confidence intervals (CI) and 95% prediction intervals (PI) were estimated using a generalized linear mixed model^11^. 95% PI represents a predicted range of the true effect in an individual or new study and provide useful information on the variability of the effect in different clinical settings^12,13^.

Outcomes from the original studies and according to RVD status were pooled and compared using random-effect models; restricted maximum likelihood was used to estimate tau for this outcome.

Pooled estimates were reported as Odds Ratios (OR) and 95% confidence intervals (CI). The inconsistency index (I^2^) was calculated to measure heterogeneity. According to pre-specified cut-offs, low heterogeneity was defined as an I^2^ of < 25%, moderate heterogeneity when I^2^ falls between 25 and 75%, and high heterogeneity when I^2^ was > 75%.

For each primary outcome, a “leave-one-out” sensitivity analysis was performed, by iteratively removing 1 study at a time to analyse their influence on pooled estimate and heterogeneity.

We also performed two subgroup analyses for the prevalence of RVD, according to the geographical location of the included studies, and the risk of bias. No subgroup analysis was performed for the all-cause death, according to the low number of studies included in this analysis. To evaluate the potential impact of COVID-19 severity on the prevalence of RVD, we also performed an univariable meta-regression analysis.

All the statistical analyses were performed using R version 4.1.0 (The R Foundation, 2021), with the use of ‘meta’, ‘metafor’ and ‘dmetar’ packages.

## Results

A total of 350 studies were retrieved from the literature search (146 from MEDLINE and 204 from EMBASE). After the selection process, a total of 29 articles were included in the analysis^14–42^ (Fig. S1 in Supplementary Materials).

### Systematic review of the included studies

Table 1 shows the baseline characteristics of the studies included in the meta-analysis. 29 studies reported data about the prevalence of RVD^14–42^, while 7 reported about all-cause mortality according to RVD status^17,19,20,30,33,35,36^. 11 studies were held in Europe^16,18–21,33–36,38,40^, 8 in North America^23,25,26,28,29,37,41,42^, 3 in Asia^30–32^, and 7 in other geographical locations^14,15,17,22,24,27,39^, including 2 multinational studies^24,27^. Cohorts included were quite homogeneous in terms of mean age of the included patients (ranging from 52 years old to 68 years old); among sex, males were generally more represented than females (up to 84%). Among studies reporting outcome, the definition of all-cause mortality comprised ICU-death^17^, in-hospital mortality^35^, 30-day mortality^36^, 90-day mortality^19^ or unspecified all-cause death^20,30,33^.

**Table 1:** Baseline characteristics of the included studies.

Study	Geographical location	Incl. criteria	Cohort (N)	RVD (N)	Age (mean)	Males (%)	ICU (%)	HTN (%)	T2DM (%)	Definition of RVD	Outcomes	Follow up time (days)
Barman (2020)^14^	Other	COVID-19 patients	90	15	56,4	51	32	35	15	TAPSE ≤ 16 mm	Prevalence	–
Bitar (2021)^15^	Other	COVID-19 patients admitted to ICU	77	9	53*	83	100	26	32	TAPSE < 16 mm, RV S’ < 9.5 cm/s, RV FAC < 35%	Prevalence	–
Bleakley (2020)^16^	Europe	COVID-19 patients underwent mechanical ventilation	84	20	52	74	100	36	22	TAPSE < 17 mm	Prevalence	–
Calderon-Esquivel (2020)^17^	Other	COVID-19 patients admitted to ICU	30	4	59	7	100	23	13	Unclear	Prevalence, ICU mortality	NR
Ceriani (2021)^18^	Europe	COVID-19 patients admitted to medium intensity unit	55	1	58,5*	64	0	36	13	TAPSE < 17 mm, RV S’ < 9.5 cm/s, RV FAC < 35%	Prevalence	–
Chotalia (2021)^19^	Europe	COVID-19 patients with ARDS	172	87	59*	77	100	37	31	TAPSE < 17 m, RV FAC < 35%	Prevalence, all-cause death	90
D'alto (2020)^20^	Europe	COVID-19 patients	94	15	63,6	75	39	67	17	Right Ventricular-arterial uncoupling (TAPSE/PASP < 0.635 mm/mmHg)	Prevalence, all-cause death	NR
Doyen (2020)^21^	Europe	COVID-19 patients admitted to ICU	43	14	60	84	100	33	28	TAPSE < 16 mm, or RV S’ < 9,5 cm/s or RV FAC < 35%	Prevalence	–
Garcia-Cruz (2020)^22^	Other	COVID-19 patients admitted to ICU	82	22	56*	62	100	48	44	TAPSE < 17 mm	Prevalence	–
Gibson (2021)^23^	North America	COVID-19 patients admitted to ICU	32	5	56	66	100	50	41	TAPSE < 18 mm	Prevalence	–
Giustino (2020)^24^	Other	COVID-19 patients	298	62	63	67	NR	59	37	TAPSE < 17 mm or RV S′ < 9,5 cm/s	Prevalence	–
Iyengar-Kapuganti (2020)^25^	North America	COVID-19 patients	59	9	66,8	NR	NR	NR	NR	Unclear	Prevalence	–
Jain (2021)^26^	North America	COVID-19 patients admitted to ICU	52	18	59,9	60	100	69	37	Visual	Prevalence	–
Karagodin (2021)^27^	Other	COVID-19 patients	509	148	60*	NR	NR	NR	NR	RVFWS > -20%	Prevalence	–
Kim (2020)^28^	North America	COVID-19 patients	268	41	64	66	68	63	41	TAPSE < 16 mm and RV Sʹ < 10 mm/s	Prevalence	–
Krishna (2021)^29^	North America	COVID-19 patients	179	54	59.8	62	62	60	37	Visual	Prevalence	–
Li Y (2021)^30^	Asia	COVID-19 with previous CVD	89	27	66	57	22	79	19	TAPSE, RV S’, RV FAC below pre-specified cut-offs	Prevalence, All-cause mortality	NR
Li YL (2021)^31^	Asia	COVID-19 patients with ARDS	49	8	64,7	51	100	35	27	TAPSE < 17 mm	Prevalence	–
Liaqat (2021)^32^	Asia	COVID-19 patients	181	29	44,6	59	NR	18	18	TAPSE (unclear cut-off)	Prevalence	–
Moody (2020)^33^	Europe	COVID-19 patients	164	58	61	78	73	41	32	TAPSE < 17 mm, or RV FAC < 35%	Prevalence, all-cause death	31 (14- 42)
Norden (2021)^34^	Europe	COVID-19 patients admitted to ICU	31	9	58	77	100	39	16	Combined (RV Score)	Prevalence	–
Pagnesi (2020)^35^	Europe	Non-ICU COVID-19 patients	200	29	66*	65	0	42	18	TAPSE < 17 or RV S' < 9,5 cm/s	Prevalence, in-hospital mortality	9 (4–14)
Rath (2020)^36^	Europe	COVID-19 patients	98	17	68	63	NR	70	24	TAPSE < 20 mm	Prevalence, 30-day mortality	30
Schott (2020)^37^	North America	COVID-19 patients	65	18	60	58	58	58	35	Unclear	Prevalence	–
Soulat-Dufour (2021)^38^	Europe	COVID-19 patients	445	65	68	66	NR	60	29	TAPSE < 17 mm, RV S' < 9.5 cm/s, RV FAC < 35%	Prevalence	–
Szekely (2020)^39^	Other	COVID-19 patients	100	14	66,1	63	NR	57	29	TAPSE < 17 mm	Prevalence	–
Van den Heuvel (2020)^40^	Europe	COVID-19 patients	51	5	63*	8	31	41	18	TAPSE < 17 mm or RV S’ velocity < 10 cm/s	Prevalence	–
Vasudev (2020)^41^	North America	COVID-19 patients	45	5	61,4	51	NR	64	55	Unclear	Prevalence	–
Wats (2021)^42^	North America	COVID-19 patients	214	61	66,5	63	NR	68	36	Combined (Visual, RV S’)	Prevalence	–

*ARDS* acute respiratory distress syndrome, *CVD* cardiovascular disease, *HTN* hypertension, *ICU* intensive care unit, *NR* not reported, *PASP* pulmonary arterial systolic pressure, *RVD* right ventricular dysfunction, *RVEF* right ventricle ejection fraction, *RV FAC* right ventricle fractional area change, *RV FWS* right ventricle free wall strain, *RV S*’ right ventricle systolic velocity, *TAPSE* tricuspid annular plane systolic excursion, *T2DM* type 2 diabetes mellitus.

*Median.

The definition of RVD was heterogeneous across studies, both in terms of parameters and cut-offs used to defined RVD. 13 studies used a combination of several parameters^15,18,19,21,24,28,30,33–35,38,40,42^, 8 studies defined RVD according to TAPSE cut-off^14,16,22,23,31,32,36,39^, while other or unclear definitions of RVD was used in 8 studies^17,20,25–27,29,37,41^. In one study, a surrogate of Right Ventricular-Arterial uncoupling, which in turn relates the degree of RVD to the increase of pulmonary pressure, was addressed^20^. For 4 studies, we assumed RVD according to the reported TAPSE cut-offs, to mitigate heterogeneity in the RVD definition among studies^14,16,22,39^.

The risk of bias for each study was reported in Tables S2 and S3 in Supplementary Materials, respectively for studies assessing the prevalence of RVD, and for studies reporting outcomes. Among studies reporting prevalence, 13 were defined at high risk of bias, while 5 studies were defined at high risk of bias among those reporting outcomes. Selection bias and definition of RVD were the most common concerns among the included studies.

### Meta-analysis of the included studies

#### Prevalence of RVD

**Figure 1:**
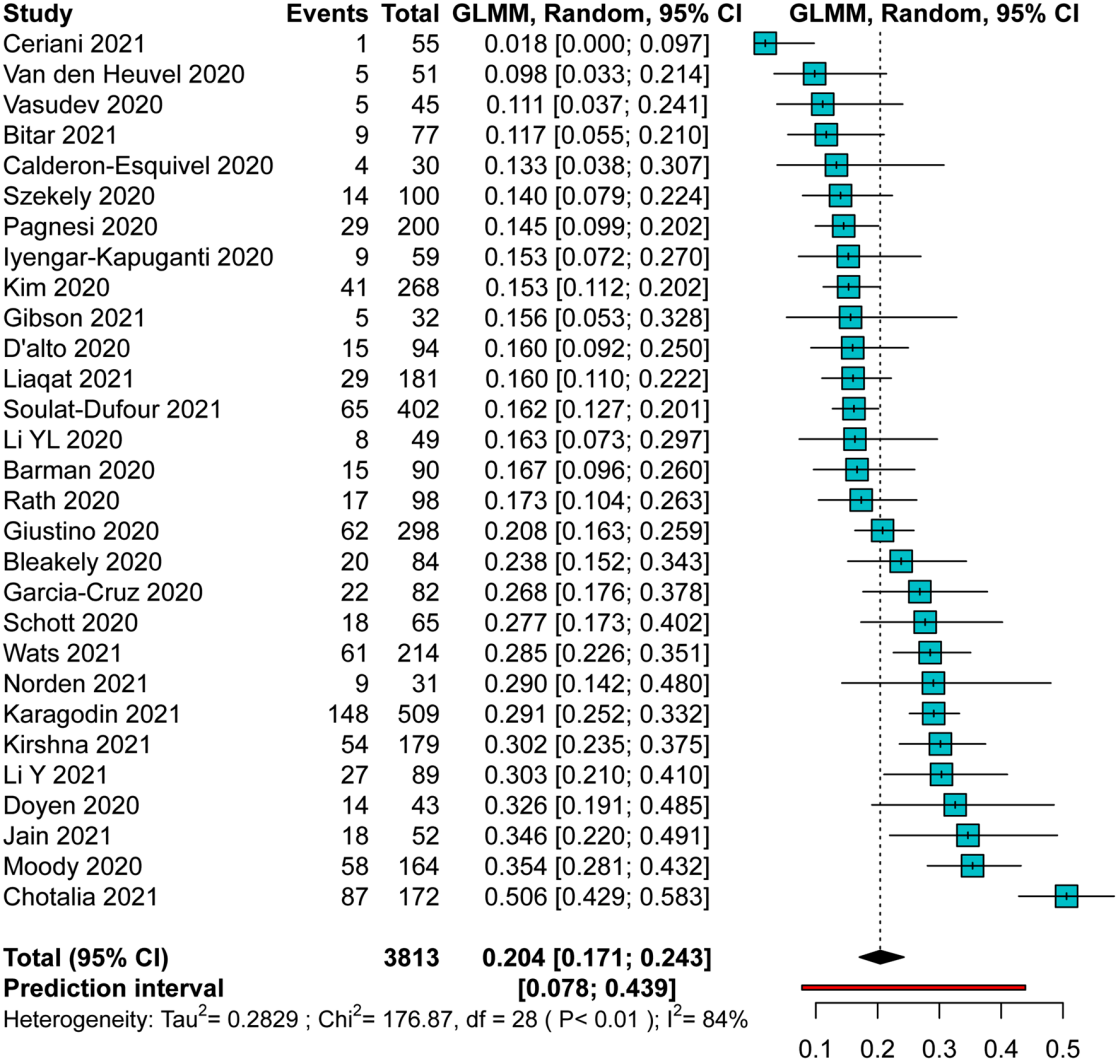
Pooled Prevalence of RVD among patients with COVID-19. *CI* confidence intervals, *GLMM* generalized linear mixed model.

Among 3813 patients included in the analysis, pooled prevalence of RVD was found as high as 20.4%, with a high degree of heterogeneity between studies. PI was between 7.8% and 43.9% (Fig. 1). No significant differences were observed in the prespecified subgroup analysis according to geographical location or bias risk (Table S4).

The prespecified leave-one-out sensitivity analysis showed overall consistency of the main results, with little to no influence of individual studies on pooled estimates or heterogeneity (Fig. S2).

**Figure 2:**
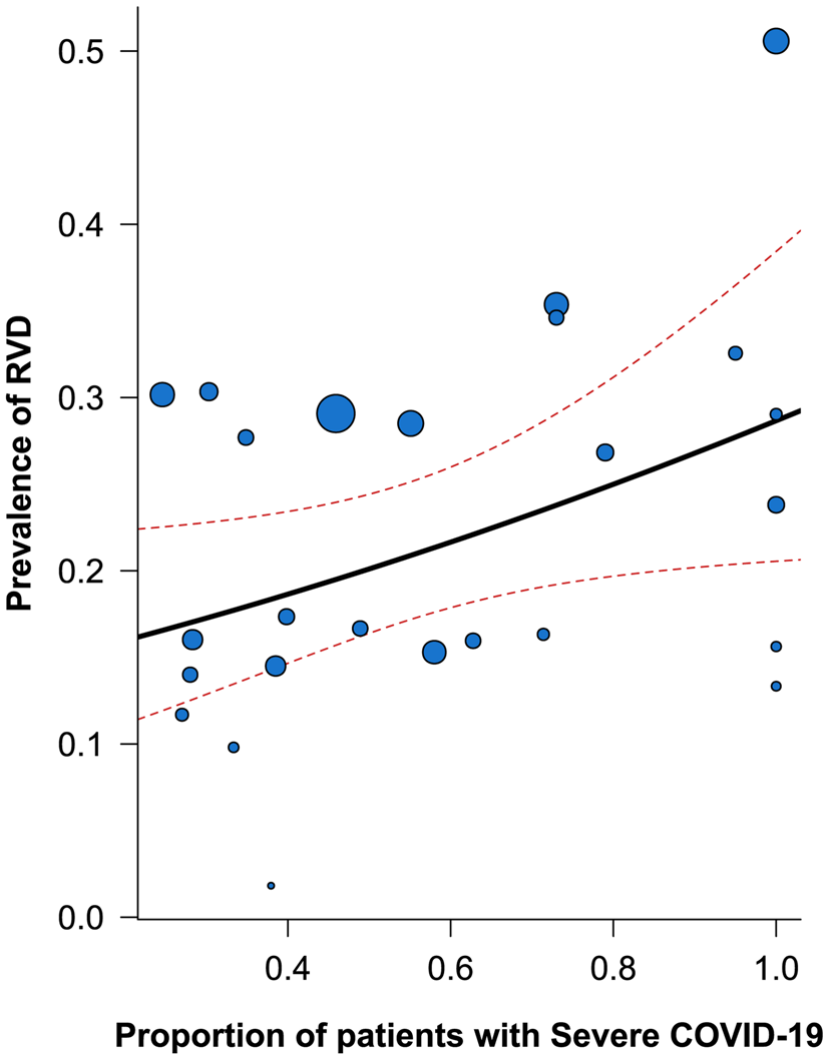
Meta-regression analysis for the prevalence of RVD according to severity of COVID-19. *RVD* right ventricular dysfunction.

To evaluate the impact of COVID-19 severity (defined as the proportion of patients defined as “severe” or “critical” in the original study, or when a stratification was not available, as those patients with ARDS or mechanically ventilated) on the prevalence of RVD, we performed an univariable meta-regression analysis. The results are graphically reported in Fig. 2; the proportion of patients with severe COVID-19 disease was significantly associated with the prevalence of RVD in the cohorts included (p = 0.040, R^2^ = 22.4%).

#### All-cause death according to RVD

**Figure 3:**
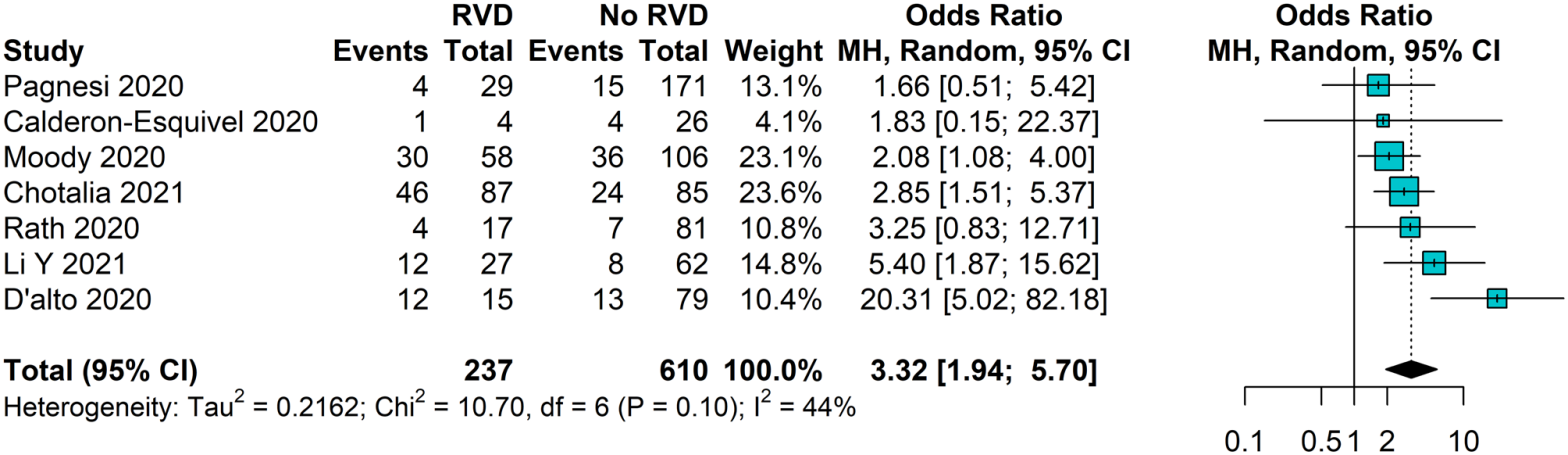
All-cause death according to RVD status in patients with COVID-19. *CI* confidence intervals; *RVD* right ventricular dysfunction.

Seven studies reported all-cause death occurrence according to RVD status, with a total of 847 patients included in the analysis. RVD was associated with a significantly increased likelihood of all-cause death (OR 3.32, 95% CI 1.94–5.70), with a moderate grade of heterogeneity between studies (Fig. 3). The leave-one-out analysis showed that excluding the study from D’Alto et al.^20^ reduces the pooled estimate, with no heterogeneity among the 6 remaining studies (I^2^ = 0%, Fig. S3). No publication bias was detected (Egger’s test p = 0.446, Fig. S4).

## Discussion

COVID-19 disease was defined as the third leading cause of death in the United States by October 2020^43^. At this stage of the pandemic, early identification of patients at higher risk of clinical deterioration is critical for proper prognostic stratification and delivering the best care. Cardiovascular complications, including myocardial dysfunction, has been described as a potential predictor of adverse outcomes^44^; although most studies focused on left ventricular impairment, some reports clearly underlines a potential detrimental role of RVD in patients with COVID-19.

Our study reports a comprehensive and updated systematic review and meta-analysis on the prevalence of RVD and associated outcomes in patients with COVID-19. Overall, we found that the prevalence of RVD may be as high as almost 1 out of 5 patients. Among the studies included, we observed a largely ranging prevalence of RVD, possibly reflecting the heterogeneity in the sensitivity of the methods used to define RVD. Bleakley et al.^16^ observed that specific phenotypes of RVD may be present in patients with COVID-19, and that definition according to TAPSE may have low sensitivity to detect RVD in this clinical scenario. The severity of the disease may also represent one key factor influencing the prevalence of RVD among COVID-19 patients, although evidence is limited. In our meta-analysis, we reported a large variation of PI, up to 44%; this information may be particularly useful to interpret the findings of our study: our results indicates that, depending on the method used to define RVD and the clinical setting, the prevalence of RVD in patients with COVID-19 may be higher than expected. This hypothesis is confirmed by our meta-regression, which found that the proportion of severe COVID-19 patients enrolled was a significant predictor for higher prevalence of RVD in the studies included. The relatively low R^2^ found for this association suggests that other factors are important in determining the prevalence of RVD, but we were unfortunately unable to analyze them, and to perform multivariable meta-regressions, due to data availability. Beyond that, and although this analysis has some clear limitation (the heterogeneous definition of severe disease, and the study-level nature of this association), the results of our meta-regression may support a mechanistic link between severe disease and RVD. However, further studies are needed to confirm this association, and to evaluate the impact of other risk factors on the risk of RVD in COVID-19 patients.

Several physiopathological hypotheses sustain association between COVID-19 and RVD. COVID-19 related ARDS is likely to be often complicated by RVD, given the direct alveolar injury and the associated ventilatory strategies such as hyper-inflated lungs and permissive hypercapnia^45^. Moreover, a direct detrimental viral effect on pulmonary microcirculation up to a pattern of endothelium with endothelial dysfunction^46^ and increased vascular inflammatory infiltrate was reported in autopsies from COVID-19 patients^3^. As a matter of fact, an interplay between COVID-19, angiotensin-converting enzyme 2, and pulmonary hypertension have been postulated^47,48^.

Furthermore, COVID-19 has been linked to an increased risk of venous thromboembolism (VTE) and pulmonary embolism^49^, with the highest risk for patients with severe disease; moving from these evidence, VTE may represent a critical cause of deterioration of RV function and performance^50^. Taken all pathophysiological mechanisms together (parenchymal involvement, endothelial damage, and pulmonary embolism), right ventricular overload with increased pulmonary pressures is likely to occur frequently. Furthermore, COVID-19 is associated with direct myocardial injury through many different mechanisms, including inflammation, microvascular dysfunction, hypoxia, and ischemia^44^, with also a COVID-19 related myocarditis described^51^. Although these manifestations may be more frequently causing left ventricular dysfunction, it is possible that they also have a role, although often overlooked, in the onset of RVD.

Beyond these hypotheses, our findings demonstrated that patients with COVID-19 and RVD are exposed to an excess of mortality than patients without RVD. Our results are in line to what has been observed in other clinical settings characterized by respiratory infectious diseases; indeed, reduced right ventricular function was reported as a risk factor for adverse events in community-acquired pneumonia^52^, as well as in patients with ARDS^45^. This information may be crucial for clinicians dealing with patients with COVID-19. In fact, bedside ultrasound examination has become increasingly important in this pandemic, for the assessment of disease progression, lung-heart interactions, and hemodynamic instability^53^, in a context where access to second-line diagnostics tools is often reduced by logistic constraints or severity of the disease, as in the critically ill patients. According to our data, careful assessment of RV function, which is often undervalued and overlooked in a fast approach to cardiac ultrasound, may provide useful information and may drive specific therapeutic approaches. Since most of the included cohorts reported about patients in ICU settings, these findings may not be immediately translated to all patients with COVID-19; however, further studies are required to confirm these results, and to explore whether a standardized screening program for RVD dysfunction, as well as tailored therapeutic approaches, may significantly improve the prognosis of these patients.

### Limitations

This study has several limitations. First, most of the studies included in this meta-analysis are retrospective or based on small cohorts, some of which were found at high risk of bias. This limitation, which may have affected our results and estimates, is mainly due to the nature of the research question, and the pandemic scenario in which these studies were conducted. Moreover, the heterogeneity in the definition of RVD may have distorted our estimates on the pooled prevalence of the disease. To overcome these limitation (at least partially), we reported PI, which gave a broader sense of the possible distribution of the actual prevalence in patients with COVID-19. Furthermore, our leave-one-out analysis showed consistency of our results after excluding one study at a time. Our study was not designed to assess factors that may influenced the association between COVID-19 and RVD; also, some baseline characteristics were missing or not reported in the original studies, and most studies did not provide details on the severity or grade of RVD. This limited our ability to evaluate the influence of specific variables on the prevalence of RVD, or stratify our results according to RVD grading. We attempted to estimate the impact of COVID-19 severity on RVD prevalence through a meta-regression analysis, which may help in understanding the association between severity of COVID-19 and burden of RVD. However, we were not able to perform multivariable meta-regression with other risk factors, due to data availability; therefore, the results of the meta-regression analysis should be interpreted with caution. Most of the patients were recruited in ICUs and/or underwent mechanical ventilation, and these factors may have influenced the assessment of RVD in the original studies and, in turn, our results. However, these patients represent a relevant part of individuals with COVID-19, so that these findings are highly relevant to everyday practice. We were only able to analyse the association between RVD and all-cause mortality in COVID-19 patients, since the original studies did not report sufficient data on the causes of death observed. Further studies are required to analyse the impact of RVD on different cause of mortality, including cardiovascular and COVID-19 related mortality.

## Conclusion

Among patients with COVID-19, RVD can be found in almost 1 out of 5 patients; the prevalence may be influenced by the severity of COVID-19 disease, but these results need confirmation in further studies. Patients with RVD showed a threefold higher likelihood of all-cause death, compared to patients with normal RV function. RVD may represent one important and often overlooked marker for prognostic stratification in COVID-19; further studies are needed to clarify this association and investigate the specific management and therapeutic approach for these patients.

## Supplementary Information

Supplementary Information.
