# Supplementary material for: Prevalence of right ventricular dysfunction and impact on all-cause death in hospitalized patients with COVID-19: a systematic review and meta-analysis
Source: Sci Rep. 2021 Sep 7;11:17774. doi: 10.1038/s41598-021-96955-8 (PMC8423751; doi:10.1038/s41598-021-96955-8)
Supplement: Supplementary file 1 — Supplementary Information. [file 41598_2021_96955_MOESM1_ESM.docx]

**Prevalence of Right Ventricular Dysfunction and Impact on All-Cause Death in hospitalized patients with COVID-19: A Systematic Review and Meta-Analysis**

Short Title: RVD in COVID-19

Bernadette Corica^1^ MD*, Alberto Maria Marra^2,3^ MD*, PhD, Stefania Basili^1^ MD,
Roberto Cangemi^1^ MD, PhD, Antonio Cittadini^2^ MD, Marco Proietti^4,5,6^ MD^†^, PhD, Giulio Francesco Romiti^1^ MD^†^

**Supplementary Materials**

**Index of Supplementary Materials**

**Table S1 -** Full Search Strategy

**Table S2 -** Bias Assessment (Newcastle Ottawa Scale) for Prevalence of Right Ventricular Dysfunction

**Table S3 –** Bias Assessment (Newcastle Ottawa Scale) for All-Cause Death according to Right Ventricular Dysfunction status

**Table S4 –** Subgroup Analysis for Right Ventricular Dysfunction Prevalence

**Figure S1 –** PRISMA Flow-Chart

**Figure S2 –** Leave-one-out analysis for Prevalence of Right Ventricular Dysfunction

**Figure S3 –** Leave-one-out analysis for All-cause Death according to Right Ventricular Dysfunction status

**Figure S4 –** Funnel Plot for All-cause Death according to Right Ventricular Dysfunction status

**Table S1 – Full Search Strategy**

| **MEDLINE** |
| --- |
| 1. "ventricular dysfunction, right"[MeSH Terms] OR ("ventricular"[All Fields] AND "dysfunction"[All Fields] AND "right"[All Fields]) OR "right ventricular dysfunction"[All Fields] OR ("right"[All Fields] AND "ventricular"[All Fields] AND "dysfunction"[All Fields]) 2. "TAPSE"[All Fields] 3. "fractional area change"[All Fields] 4. #1 OR #2 OR #3 5. "severe acute respiratory syndrome coronavirus 2"[Supplementary Concept] OR "severe acute respiratory syndrome coronavirus 2"[All Fields] OR "ncov"[All Fields] OR "2019-nCoV"[All Fields] OR "COVID-19"[All Fields] OR "SARS-CoV-2"[All Fields] OR ((coronavirus[All Fields] OR "cov"[All Fields])) 6. "sars-cov-2"[MeSH Terms] OR "sars-cov-2"[All Fields] OR "sars cov 2"[All Fields] 7. "Covid-19"[All Fields] OR "Covid"[All Fields] 8. #5 OR #6 OR #7 9. #4 AND #8 |
| **EMBASE** |
| 1. right AND ventricular AND dysfunction 2. tapse 3. 'fractional area change' 4. #1 OR #2 OR #3 5. 'coronavirus disease 2019'/exp 6. Covid 7. 'severe acute respiratory syndrome coronavirus 2’ 8. 'sars-cov-2' 9. #5 OR #6 OR #7 OR #8 10. #4 AND #9 |

**Table S2 -** **Bias Assessment (Newcastle Ottawa Scale) for Prevalence of Right Ventricular Dysfunction**

| **Article** | **Selection (2)** | **Comparability (1)** | **Endpoint (2)** | **Total (5)** | **Risk** |
| --- | --- | --- | --- | --- | --- |
| **Barman 2020*** | 1 | 1 | 2 | 4 | **Low** |
| **Bitar 2021** | 2 | 1 | 2 | 5 | **Low** |
| **Blakely 2020#** | 2 | 0 | 2 | 4 | **Low** |
| **Calderon-Esquivel 2020°**§ | 1 | 1 | 1 | 3 | **High** |
| **Ceriani 2021#** | 2 | 0 | 2 | 4 | **Low** |
| **Chotalia 2021*#** | 1 | 0 | 2 | 3 | **High** |
| **D'alto 2020°** | 2 | 1 | 1 | 4 | **Low** |
| **Doyen 2020***§ | 0 | 1 | 2 | 3 | **High** |
| **Garcia-Cruz 2020**ç | 2 | 1 | 1 | 4 | **Low** |
| **Gibson 2021*** | 0 | 1 | 2 | 3 | **High** |
| **Giustino 2020**#ç | 2 | 0 | 1 | 3 | **High** |
| **Iyengar-Kapuganti 2020**°#ç | 2 | 0 | 0 | 2 | **High** |
| **Jain 2021°*** | 1 | 1 | 1 | 3 | **High** |
| **Karagodin 2021#**ç | 2 | 0 | 1 | 3 | **High** |
| **Kim 2020**# | 2 | 0 | 2 | 4 | **Low** |
| **Krishna 2021°** | 2 | 1 | 1 | 4 | **Low** |
| **Li Y 2021*#** | 1 | 0 | 2 | 3 | **High** |
| **Li YL 2021***§° | 0 | 1 | 2 | 3 | **High** |
| **Liaqat 2021**#° | 2 | 0 | 1 | 3 | **High** |
| **Moody 2020** | 2 | 1 | 2 | 5 | **Low** |
| **Norden 2021**§* | 0 | 1 | 2 | 3 | **High** |
| **Pagnesi 2020** | 2 | 1 | 2 | 5 | **Low** |
| **Rath 2020°** | 2 | 1 | 1 | 4 | **Low** |
| **Schott 2020°** | 2 | 1 | 1 | 4 | **Low** |
| **Soulat-Dufour 2021** | 2 | 1 | 2 | 5 | **Low** |
| **Szekely 2020**ç | 2 | 1 | 1 | 4 | **Low** |
| **Van den Heuvel 2020** | 2 | 1 | 2 | 5 | **Low** |
| **Vasudev 2020**ç§ | 1 | 1 | 1 | 3 | **High** |
| **Wats 2021°** | 2 | 1 | 1 | 4 | **Low** |

*Selected cohort

§ Sample size < 50 patients

# Some patients not evaluated and not defined

° Measurement for RVD not defined

ç Incompletely reported descriptive statistics

**Table S3 – Bias Assessment (Newcastle Ottawa Scale) for All-Cause Death according to Right Ventricular Dysfunction status**

| **Studies** | **Selection (4)** | **Comparability (2)** | **Endpoint (4)** | **Total (9)** | **Risk** |
| --- | --- | --- | --- | --- | --- |
| **Calderon-Esquivel 2020°***§ | 2 | 2 | 2 | 6 | **High** |
| **Chotalia 2021**#§ | 3 | 1 | 2 | 6 | **High** |
| **D'alto 2020°***§ | 2 | 2 | 2 | 6 | **High** |
| **Li Y 2020**#*§ | 3 | 1 | 2 | 6 | **High** |
| **Moody 2020*** | 4 | 2 | 3 | 9 | **Low** |
| **Pagnesi 2020***§ | 4 | 2 | 2 | 8 | **Low** |
| **Rath 2020°***#§ | 2 | 2 | 1 | 5 | **High** |

° Measurement for RVD not defined

* Endpoint defined with medical records

# Non-responders not described
§ Endpoint not blinded

**Table S4 – Subgroup Analysis for Right Ventricular Dysfunction Prevalence**

| **Subgroups** | **N° Studies** | **Pooled Prevalence** | **95% CI** | **I^2^** |
| --- | --- | --- | --- | --- |
| *Geographical Location (p for subgroup differences=0.919)* | | | | |
| **Europe** | 11 | 20.1 | 13.3-29.1 | 91.2 |
| **North America** | 8 | 22.1 | 16.9-28.4 | 75.0 |
| **Asia** | 3 | 20.4 | 13.9-28.9 | 74.6 |
| **Other** | 7 | 19.4 | 14.8-25.0 | 75.8 |
| *Risk of Bias (p for subgroup differences=0.155)* | | | | |
| **Low Risk** | 16 | 18.4 | 14.6-22.9 | 80.2 |
| **High Risk** | 13 | 23.6 | 18.1-30.1 | 85.2 |

**Legend:** CI= Confidence Interval; RVD= Right Ventricular Dysfunction.

**Figure S1 – PRISMA Flow-Chart**


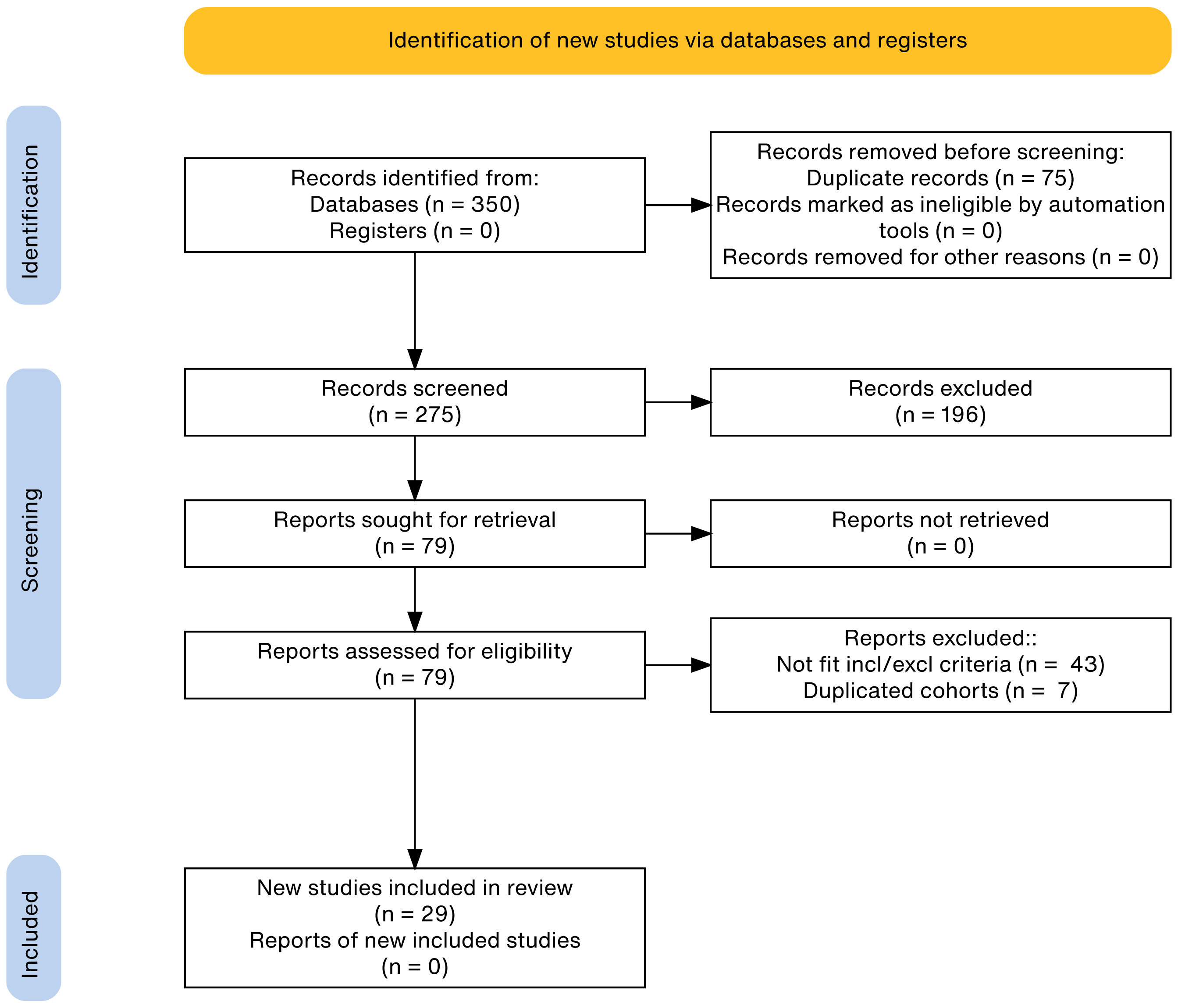


**Figure S2 – Leave-one-out analysis for Prevalence of Right Ventricular Dysfunction**

**
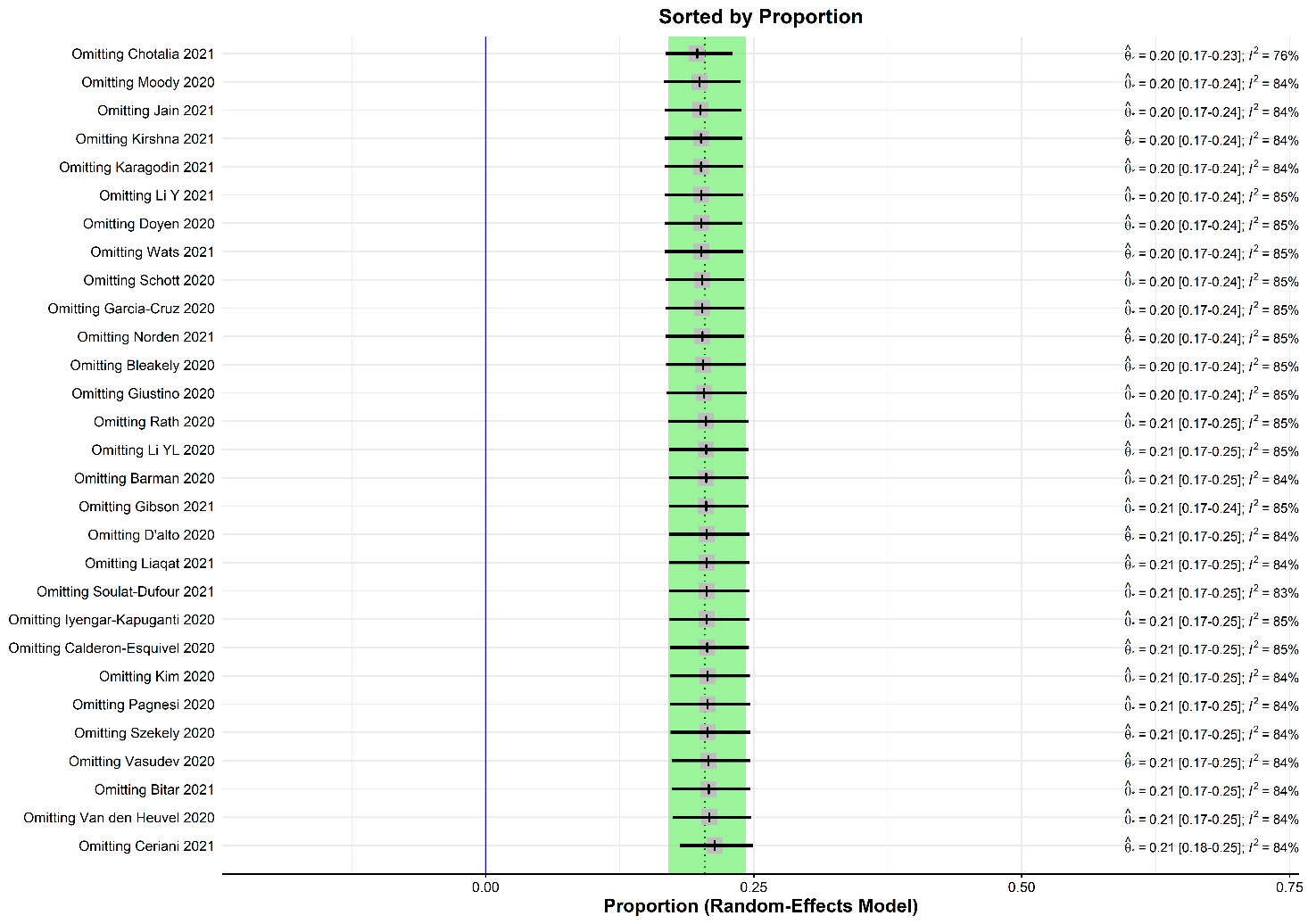
**

**Figure S3 – Leave-one-out analysis for All-cause Death according to Right Ventricular Dysfunction status**

**
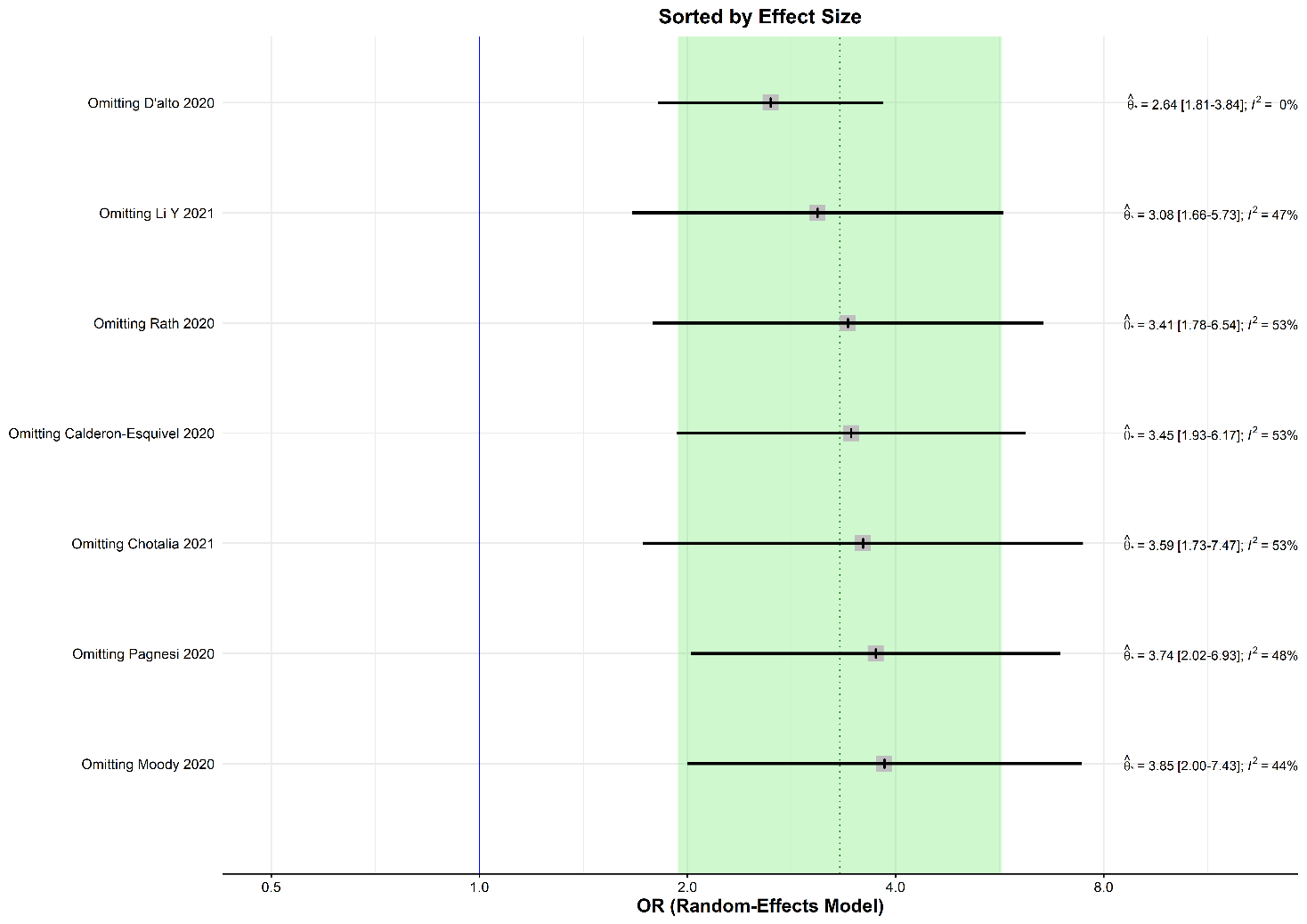
**

**Figure S4 – Funnel Plot for All-cause Death according to Right Ventricular Dysfunction status**

**
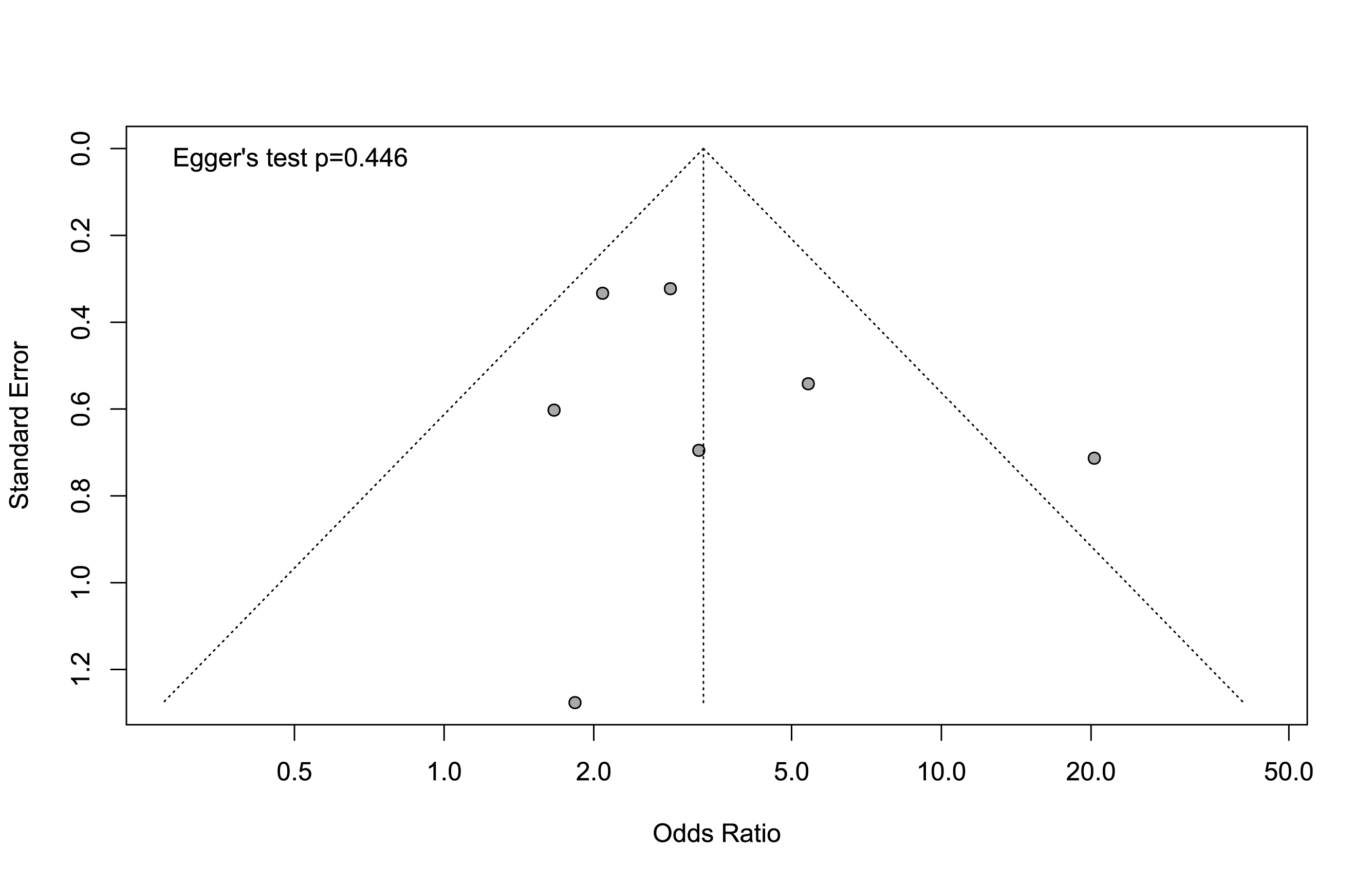
**
